# Anthranilic Acid–G-Protein Coupled Receptor109A–Cytosolic Phospholipase A2–Myelin–Cognition Cascade: A New Target for the Treatment/Prevention of Cognitive Impairment in Schizophrenia, Dementia, and Aging

**DOI:** 10.3390/ijms252413269

**Published:** 2024-12-10

**Authors:** Gregory Oxenkrug

**Affiliations:** Department of Psychiatry, Tufts University School of Medicine, Boston, MA 02111, USA; gregory.oxenkrug@tufts.edu

**Keywords:** anthranilic acid, Alzheimer’s dementia, cognition, cytosolic phospholipase A2, G-protein coupled receptor 109A, myelin, aging, schizophrenia, sex-specific effect

## Abstract

Cognitive impairment is a core feature of neurodevelopmental (schizophrenia) and aging-associated (mild cognitive impairment and Alzheimer’s dementia) neurodegenerative diseases. Limited efficacy of current pharmacological treatments warrants further search for new targets for nootropic interventions. The breakdown of myelin, a phospholipids axonal sheath that protects the conduction of nerve impulse between neurons, was proposed as a neuropathological abnormality that precedes and promotes the deposition of amyloid-β in neuritic plaques. The present review of the recent literature and our own pre- and clinical data suggest (for the first time) that the anthranilic acid (AA)-induced activation of microglial-expressed G-protein coupled receptor (GPR109A) inhibits cytosolic phospholipase A2 (cPLA2), an enzyme that triggers the degradation of myelin and consequently attenuates cognitive impairment. The present review suggests that the up-regulation of AA formation is a sex-specific compensatory (adaptive) reaction aimed to prevent/treat cognitive impairment. The AA–GPR109A–cPLA2–myelin–cognition cascade suggests new nootropic interventions, e.g., the administration of pegylated kynureninase, an enzyme that catalyzes AA formation from Kynurenine (Kyn), a tryptophane catabolite; pegylated interferon-alpha; central and peripheral Kyn aminotransferase inhibitors that increase availability of Kyn as a substrate for AA formation; and vagus nerve stimulation. The cascade predicts nootropic activity of exogenous GPR109A agonists that were designed and underwent clinical trials (unsuccessful) as anti-dyslipidemia agents. The proposed cascade might contribute to the pathogenesis of cognitive impairment. Data on AA in neurodegenerative disorders are scarce, and the proposed cascade needs further exploration in pre- and clinical studies

## 1. Introduction

Cognitive impairment is a core feature of neurodevelopmental (e.g., schizophrenia) and aging-associated neurodegenerative diseases, such as mild cognitive impairment (MCI) and Alzheimer’s dementia (AD) [1,2,3]. The limited efficacy of current pharmacological treatments, which are based on monoamines and amyloid-β (Aβ) models, underscores the need for the further exploration of new targets for nootropic interventions and mechanisms of cognitive impairment. According to the myelin hypothesis, a deficit in myelination precedes Aβ deposition in the human brain and underlies cognitive impairment associated with schizophrenia, MCI, AD, and aging [4]. The present review discusses, for the first time, the impact of anthranilic acid (AA), a putative endogenous agonist of the G-protein coupled receptor (GPR109A), on myelin integrity and, consequently, on the prevention and treatment of cognitive impairment.

## 2. Myelin Hypothesis of Cognitive Impairment

Oligodendrocytes are responsible for the production of myelin, an electrically insulating phospholipid sheath composed of up to 100 layers tightly wound on top of each other around axons. Myelination results in a 3000-fold increase in the information processing capacity of the brain’s “internet” [5,6]. Myelin’s “insulating” role is essential for normal cognitive, sensory, and motor functions. Myelination begins during the last trimester of human pregnancy and continues through childhood, adolescence, and into adulthood. The myelination of regions associated with early life functions, such as motor and sensory systems, begins earlier than that of regions critical for later life functions, such as language and executive and global cognitive functioning. AD pathology develops earlier in brain regions that myelinate later than in those that myelinate earlier [7]. Myelin breakdown in the late-myelinating brain regions is suggested to be the first neuropathological abnormality that precedes and promotes the deposition of Aβ in neuritic plaques [1]. Experimental studies revealed that myelin deterioration begins earlier in 5XFAD mice (an AD mouse model) than in wild-type mice at one and five months of age, respectively, and is associated with spatial memory learning deficits [8]. Notably, myelin deterioration precedes the onset of the increased Aβ load observed in two-month-old 5XFAD mice [9]. In human brains, myelin damage also precedes Aβ deposition [10]. Decreased myelination accelerates cognitive decline among cognitively unimpaired individuals [11], is associated with cognitive dysfunction in individuals with schizophrenia [12], and is linked to major risk factors for AD, such as aging and female sex [13]. The myelin sheath should remain intact to support the integration of information across the neural networks that support cognitive and motor functions [14]. The high prevalence of myelin degradation over myelin biosynthesis might result in a deficit in myelination, which is associated with cognitive impairment in neurodegenerative diseases [15].

## 3. Cytosolic Phospholipase A2 and Myelin Degradation

The activity of cytosolic phospholipase A2 (cPLA2) is a (mostly overlooked) mechanism that affects myelin degradation [16]. Blocking cPLA2 markedly slows myelin degradation in the distal nerve segment of the transected sciatic nerve in C57BL/6 mice [17]. Elevated cPLA2 activity has been reported in schizophrenia-specific brain areas in subjects at high risk for the development of psychosis [18] and AD [19]. The activation of cPLA2 triggers the hydrolysis of myelin into phosphates and free fatty acids (FFAs). FFA levels reflect myelin degradation activity in the brain and are used in clinical practice as an index of cPLA2 activity. Plasma/serum FFA levels are elevated in individuals with AD [7] and schizophrenia, while a decline in FFA levels correlates with clinical improvement in schizophrenia patients [20].

An increase in glucose concentrations in the medium elevates cPLA2 activity in cultured rat and human vascular smooth muscle cells and in rat capillary endothelial cells [21], suggesting that insulin resistance contributes to cognitive impairment via cPLA2activation and the consequent augmentation of myelin degradation.

Notably, N-acetyl-serotonin (NAS), an immediate precursor of melatonin and a food supplement, exerts nootropic effects in fish [22]. 5-methoxycarbonylamino-N-acetyltryptamine (5-NAT), a putative NAS receptor agonist [23], decreases cPLA2 protein and mRNA levels in a dose-dependent manner [24]. We found that three weeks of treatment with NAS protects against neurotoxicity induced by Aβ fragment 25–35 in rat cerebellar granule cell cultures and reverses cholinergic neurotoxin-induced cognitive impairment (failure to perform tasks in the active avoidance and water maze tests) in Wistar rats [25]. Therefore, the inhibition of cPLA2 activity might contribute to the nootropic and neuroprotective effects of NAS [26,27].

## 4. G-Protein Coupled Receptor 109A Inhibits Cytosolic Phospholipase A2

The GPR109A agonists niacin and R-β-hydroxybutyric acid (BHB), ketone bodies produced by hepatocytes, acutely decrease FFA levels, which serve as an index of cPLA2 activity. In vitro studies demonstrated a decrease in FFA levels in rat primary adipocytes following treatment with niacin or BHB. Similarly, serum FFA levels decreased in wild-type mice within 10 min following niacin administration; however, niacin had no effect on FFA levels in 3T3-L1 adipocytes, which have limited cell surface expression of the GPR109A receptor, or in GPR109A receptor knockout mice [28].

## 5. GPR109A Downregulation and Cognitive Impairment

GPR109A was deorphanized in 2003 as a niacin receptor [29]. GPR109A, also known as hydroxycarboxylic acid receptor 2 (HCAR2), is highly expressed in adipocytes, macrophages, and microglia in the brain areas associated with schizophrenia [30,31,32] and AD [33]. The human GPR109A gene is located on the long arm of chromosome 12 at position 24.31 (notated as 12q2). GPR109A activation induced the downregulation of proinflammatory IL-6 cytokine formation and the upregulation of anti-inflammatory IL-10 cytokine production, which might attenuate the development of chronic low-grade inflammation associated with cognitive impairment and aging [34]. GPR109A expression decreases with aging [28] and in individuals with AD [7]. Moreover, a genome-wide study suggested a genetic mutation of GPR109A in at least one subgroup of schizophrenia [16]. Notably, the suppression of the GPR109A gene due to diet-induced obesity might contribute to the association between obesity and the risk of cognitive impairment [35].

**Figure 1:**
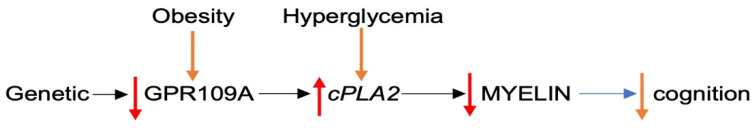
Proposed GPR109A downregulation in cognitive impairment. Abbreviations: GPR109A: G-protein coupled receptor; cPLA2: cytosolic phospholipase A2. 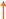 up-regulation 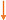 down-regulation.

The reviewed data suggest that the downregulation of GPR109A, caused by genetic (e.g., in schizophrenia) [16] and/or environmental factors (e.g., obesity in AD) [35], disinhibits cPLA2, an enzyme that triggers the degradation of myelin and, consequently, contributes to cognitive impairment in schizophrenia and aging-associated MCI and AD (Figure 1).

## 6. G-Protein Coupled Receptor 109A Agonists and Nootropic Effect

The proposed contribution of GPR109A downregulation to cognitive impairment suggests that agonists of GPR109A exert a nootropic effect.

Preclinical studies have demonstrated that GPR109A agonists, such as niacin and R-β-hydroxybutyric acid (BHB), attenuate disease progression and the accumulation of Aβ into senile plaques and improve passive avoidance behaviors and responses to the Morris water maze test in the 5XFAD mouse model of Alzheimer’s disease [8,36]. The selective induction of GPR109A in the microglia of 5xFAD mice reduces neuronal loss and plaque burden and rescues working memory deficits [9]. A high acute dose of BHB suppresses schizophrenia-like behaviors in the MK-801 model of schizophrenia, including MK-801-induced locomotor hyperactivity and the disruption of prepulse inhibition (PPI). Chronic BHB treatment attenuates MK-801-induced hyperlocomotion, and reduces sociability, and disrupts PPI [37]. Notably, the anti-inflammatory and neuroprotective effects of niacin and BHB were abolished in GPR1109A (-/-)- and siRNA-treated animals [38].

Clinical studies have revealed the nootropic effect of the GPR109A agonists niacin and BHB in individuals with schizophrenia and AD. Niacin intake had a protective effect on the development of AD and cognitive decline in a study conducted from 1993 to 2002 in a geographically defined Chicago community of 6158 residents aged 65 years and older who were initially unaffected by AD. Cognitive tests were administered to all study participants at 3-year intervals during a 6-year follow-up. Niacin intake was inversely associated with AD (*p* for linear trend = 0.002), with a higher intake of niacin associated with a slower annual rate of cognitive decline [39]. Furthermore, the serum levels of BHB in 38 schizophrenia patients of Han Chinese ethnicity were higher than in 38 healthy control subjects and were significantly correlated with executive function (r = 0.424; *p* = 0.008) [40].

## 7. Anthranilic Acid as a Putative G-Protein Coupled Receptor 109A Agonist

Dyslipidemia was an initial clinical target of niacin. Concentrations of endogenous GPR109A agonists, such as niacin, BHB, and gut microbiome-derived butyric acid (BA) [41], do not reach sufficient levels to activate GPR109A under physiological conditions. Furthermore, they are not selective agonists of GPR109A but exert low affinity for GPR109B as well. Only mega doses of niacin were clinically effective in the treatment of dyslipidemia; however, mega doses are prone to side effects. For instance, BHB might accelerate colorectal cancer proliferation [42]. Therefore, there was a need for new selective GPR109A agonists; such agonists were discovered among AA derivatives [43]. The AA molecule shares a carboxylic group moiety with other GPR109A agonists. The presence of carboxylic acid moiety in AA, niacin, and in the synthetic GPR109A agonists is necessary for the activation of GPR109A [43]. The presence of the AA skeleton was suggested to be essential for the efficacy of cPLA2 inhibition [44]. Therefore, AA might be considered as an endogenous GPR109A agonist.

**Figure 2:**
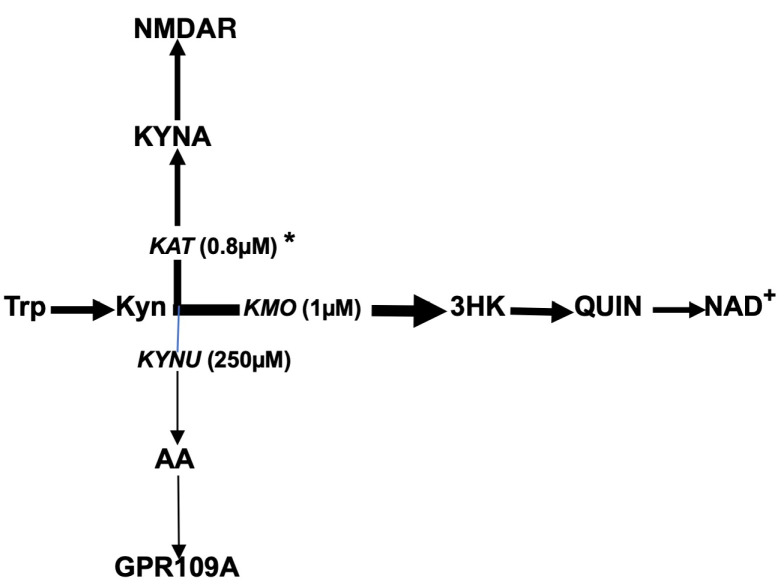
Tryptophan–kynurenine–anthranilic acid pathway in humans. Abbreviations: Trp: tryptophan; Kyn: kynurenine; 3HK: 3-hydroxykynurenine; AA: anthranilic acid; KYNA: kynurenic acid; QUIN: quinolinic acid; NAD^+^: nicotinamide adenine dinucleotide; KMO: kynurenine 3-monooxygenase; KYNU: kynureninase; KAT: kynurenine aminotransferase; GPR109A: G-protein coupled receptor; NMDAR: N-methyl-D-aspartate receptor. * denotes Michaelis–Menten constant.

The main sources of AA are the tryptophan–Kyn pathway (Figure 2) and the gut microbiome, which produces AA as a precursor for tryptophan biosynthesis [45]. Tryptophan conversion into Kyn is catalyzed by stress hormone-activated hepatic tryptophan 2,3 dioxygenase 2 (TDO) or by proinflammatory cytokine-induced extrahepatic (including microglia) indoleamine 2,3-dioxygenase (IDO). Kyn downstream catabolism is trifurcated into the formation of 3-hydroxykynurenine (3HK), kynurenic acid (KYNA), and anthranilic acid (AA), catalyzed by kynurenine 3-monooxygenase (KMO), kynurenine aminotransferase (KAT), and kynureninase, respectively [46,47,48]. A comparison of Michaelis–Menten constants for KMO, KAT, and kynureninase suggests the preferential conversion of Kyn into 3HK and KYNA rather than into AA under physiological conditions [49]. Notably, of the three enzymes that use Kyn as a substrate, KAT and kynureninase are unsaturated enzymes that are able to utilize the increased availability of Kyn resulting from KMO deficiency (e.g., in the brains of individuals with schizophrenia) [50]. Therefore, the KYNA hypothesis suggests that the downstream catabolism of Kyn in schizophrenia is shifted from the formation of 3HK toward the elevated production of KYNA, an NMDA antagonist, and that the upregulated formation of KYNA is causatively linked to the major psychopathology of schizophrenia [51,52].

Animal studies have shown that the impairment of KMO, as observed in the brains of individuals with schizophrenia, results in the upregulation of both KYNA and AA formation. KMO inhibition results in a 7-fold increase in brain AA (from 9.51 ± 1.2 to 73 ± 14 pmol/g) and a 3-fold elevation of KYNA content, from 99 ± 24 to 298 ± 40 pmol/g, confirming that when KMO is inhibited, Kyn metabolism occurs mainly through the metabolic pathways catalyzed by kynureninase or KAT [53]. Moreover, the plasma levels of AA were significantly (and independently of anti-psychotic administration) elevated in rats subjected to post-weaning social isolation rearing, a putative animal model of schizophrenia [54].

## 8. Assessment of Anthranilic Acid in Clinical Settings

Unlike KYNA, peripherally originating AA is transported into the brain through the blood–brain barrier (BBB) [55]. Notably, plasma concentrations of KYN, 3HK, and AA significantly correlated with the corresponding CSF levels [55,56]. Therefore, the evaluation of circulating levels of AA might be useful for assessing brain AA metabolism [57,58].

## 9. Anthranilic Acid in Schizophrenia

In our pilot study, we found significantly higher plasma AA levels in individuals with schizophrenia than in healthy subjects [59]. Furthermore, serum AA levels were elevated in individuals with first and multiple episodes of schizophrenia but not in their first-degree relatives [60]. However, these studies did not assess sex-specific differences in AA circulating levels or their correlation with the severity of clinical symptoms. Further studies in acutely sick, non-treated individuals with schizophrenia revealed higher (by 27%) AA plasma levels in females (n = 20) than in males (n = 31). Moreover, the plasma AA levels of female (but not male) patients before treatment positively correlated with the general psychopathology subscale score of PANSS (r = 0.625, *p* < 0.019, Spearman’s test) and predicted PANSS scores: PANSS = 11.11 + 0.94 AA [61]. After six weeks of treatment with anti-psychotics, AA plasma levels were inversely correlated (r = −0.65, *p* < 0.002, n = 20) with the severity of schizophrenia symptoms in responders (defined as a 50% reduction in PANSS total scores) but not in non-responders (defined as less than a 50% improvement) (R = −0.125, *p* = 0.63, n = 19). Post-treatment plasma AA levels were not different between female and male patients [62]. The positive correlation of AA with PANSS (i.e., the association of high AA levels with more severe schizophrenia symptoms) before treatment may suggest that AA elevation contributes to the development of schizophrenia, similar to the elevation of KYNA, an antagonist to NMDAR (50–52). Alternatively, the change from positive (pre-treatment) to inverse (post-treatment) correlation between AA and PANSS (i.e., the association of high AA levels with less severe post-treatment schizophrenia symptoms) might suggest that the pre-treatment AA elevation in female individuals with schizophrenia is an adaptive and compensatory mechanism aimed to limit the schizophrenia process.

## 10. Anthranilic Acid in Alzheimer’s Dementia

Higher plasma levels of AA (but not tryptophan, 3HK, KYNA, Kyn, quinolinic acid, xanthurenic acid, or the two hundred and seventeen other studied metabolites) were associated with a greater risk of dementia. The risk increased by 40% for each standard deviation of AA plasma levels in a study of 2067 dementia-free participants (mean age of 55.3 ± 9.5; 52% women) who were followed over an average period of 15.8 ± 5.2 years, during which 93 developed incident dementia [63]. Furthermore, AA serum levels were higher in cognitively unaffected female (but not male) subjects with a high neocortical amyloid-β load (NAL+), as measured using positron emission tomography, than in NAL− subjects, and AA serum levels also predicted NAL+ (*p* = 0.005) [64].

## 11. Anthranilic Acid in Post-Stroke Cognitive Impairment

High plasma AA levels were associated with better episodic memory in 198 stroke patients aged 65.4 ± 10.8 years. A similar but non-significant trend was observed for working memory [65].

## 12. Upregulation of Anthranilic Acid Formation in Context of Cognitive Impairment

**Figure 3:**
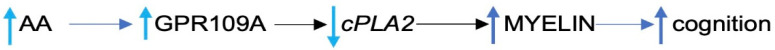
Proposed mechanism of nootropic effect of anthranilic acid. Abbreviations: AA: anthranilic acid; GPR109A: G-protein coupled receptor; cPLA2: cytosolic phospholipase 2. 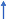 Up-regulation 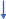 Down-regulation.

The present review suggests that upregulating the formation of AA, a putative endogenous GPR109A agonist, is a compensatory (adaptive) response aimed at attenuating the development of cognitive impairment in schizophrenia, MCI, and AD (Figure 3). Down-streaming signaling pathways following the upregulation of AA formation include the activation of G-protein coupled receptor109A, the inhibition of cytosolic phospholipase A2, and the attenuation of myelin degradation. Consequences of myelin deficiency on neuronal functioning, including cognitive functioning, were reviewed in Section 2.

## 13. Key Therapeutic Potentials of Modulation of Anthranilic Acid–G-Protein Coupled Receptor109A–Cytosolic Phospholipase A2–Myelin Cascade for Improvement of Cognitive Function in Schizophrenia, Dementia, and Aging

### 13.1. Interventions Aimed at Upregulation of Endogenous Anthranilic Acid Formation

In the context of the proposed cascade, upregulating the formation of AA, a putative endogenous GPR109A agonist, contributes to the prevention/treatment of cognitive impairment. AA formation from Kyn is catalyzed by pyridoxal-5′-phosphate-dependent kynureninase (EC 3.7.1.3). The predicted amino acid sequence of human kynureninase displayed a high similarity to that reported for the rat enzyme [66]. All tested mammalian livers (mouse, guinea pig, dog, and human) contained high kynureninase activity, while the kidneys, spleen, lung, brain, heart, and muscle had much lower activity [67]. Therefore, most of the AA originates in the peripheral organs and is transported into the brain via the BBB [57].

### 13.2. Pegylated Human Kynureninase

Pegylated human kynureninase activates Kyn conversion into AA [68] and inhibits large B16-F10 tumor growth in C57B6L/6j mice [69]. However, it has not yet been tested as a nootropic agent in the experimental models of neurodegenerative diseases.

### 13.3. Interferons

Stimulation with interferon-gamma (IFN-γ) substantially increased indoleamine 2,3-dioxygenase and kynureninase activities in primary peripheral blood macrophages and fetal brains (astrocytes and neurons), as well as in cell lines derived from macrophages/monocytes (U373MG astrocytoma), SKHEP1 liver and lung cells [70], murine cloned macrophages and microglial cells [71], human dermal fibroblasts [72], and human monocyte-derived macrophages [73].

In a prospective longitudinal study of chronic hepatitis C, AA plasma concentrations were significantly higher at week 24 in patients who received pegylated interferon-alpha for 24 weeks compared to baseline [74].

### 13.4. Sodium Benzoate and Nootropic Effect

**Figure 4:**
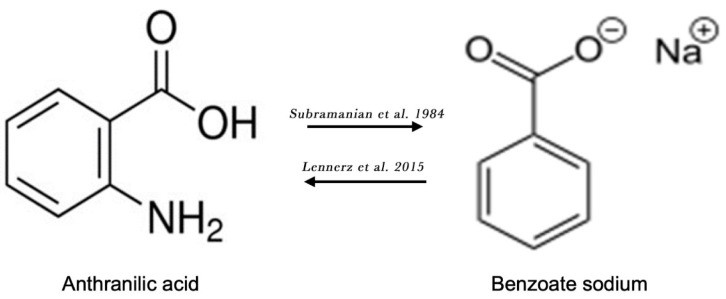
Chemical structures of anthranilic acid and benzoate sodium. Lennerz et al. [75]; Subramanian et al. [76].

Both AA (i.e., 2-Aminobenzoic acid) and sodium benzoate (an FDA approved food preservative) are derivatives of aminobenzoic acid, containing a benzene ring with an attached carboxylic group moiety (Figure 4). Notably, a robust 5-fold elevation in plasma AA levels after the administration of a single dose of sodium benzoate was an unexpected finding in a randomized, controlled cross-over clinical trial on the metabolic impact of sodium benzoate in 14 overweight but otherwise healthy human volunteers [75]. Alternatively, anthranilate hydroxylase may catalyze the conversion of AA into 2,3-dihydroxybenzoic acid via oxidative deamination and the dihydroxylation of AA (Figure 4) [76]. Therefore, AA may contribute to the clinical effects of sodium benzoate.

The nootropic effect of sodium benzoate was studied in a randomized, double-blind, placebo-controlled trial at four major medical centers in Taiwan. Sixty patients with amnestic mild cognitive impairment or mild AD were treated with 250–750 mg/day of sodium benzoate or a placebo for 24 weeks. The Alzheimer’s Disease Assessment Scale—cognitive subscale (the primary outcome) and global function (assessed via a Clinician Interview-Based Impression of Change plus Caregiver Input)—was measured every 8 weeks. Furthermore, an additional cognition composite was measured at baseline and at endpoint.

Patients treated with sodium benzoate showed an improvement in the Alzheimer’s Disease Assessment Scale, the cognitive subscale (*p* = 0.0021, 0.0116, and 0.0031 at week 16, week 24, and endpoint, respectively), the additional cognition composite (*p* = 0.007 at endpoint), and in the Clinician Interview-Based Impression of Change plus Caregiver Input (*p* = 0.015, 0.016, and 0.012 at week 16, week 24, and endpoint, respectively) compared to patients that received the placebo. Sodium benzoate was well tolerated without any evident side effects. In addition, sodium benzoate improved cognition in both female and male individuals with MCI [77]. Moreover, in a 6-week double-blind study, patients with schizophrenia who were treated with sodium benzoate (1000 mg/day) performed better in tasks assessing the speed of processing (*p* = 0.03, ES = 0.65) and visual learning and memory (*p* = 0.02, ES = 0.70) [78].

### 13.5. Inhibitors of Kynurenine Aminotransferase

**Figure 5:**
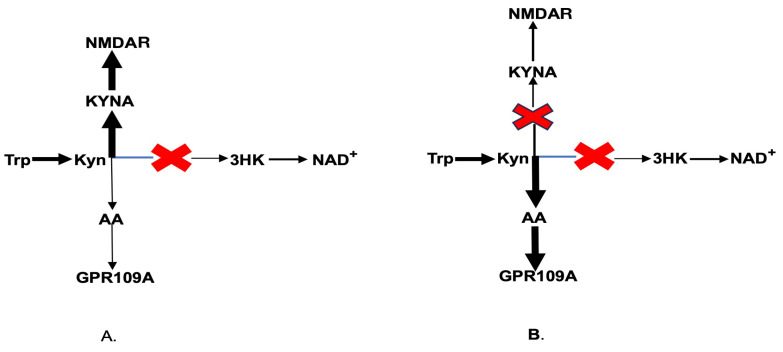
Kynurenine downstream catabolism in schizophrenia (**A**) and under KAT inhibition in schizophrenia (**B**). Abbreviations: Trp: tryptophan; Kyn: kynurenine; KYNA: kynurenic acid; 3HK: 3-hydroxykynurenine; AA: anthranilic acid; NAD+: nicotinamide adenine dinucleotide; GPR109A: G-protein coupled receptor 109A; NMDAR: N-methyl-D-aspartate receptor.

As previously described, Kyn is the common substrate for KMO, KAT, and kynureninase (Figure 2). KMO impairment in schizophrenia-specific brain areas (e.g., Brodmann area 10) increases the availability of Kyn as a substrate for both kynureninase and KAT. The elevated production of KYNA, an NMDA antagonist, was causally linked to the major psychopathology of schizophrenia. Therefore, KAT inhibitors were designed for the treatment of schizophrenia. Recently, clinical trials of a new KAT inhibitor, KYN-5356, which blocks brain KYNA formation, were initiated for the treatment of cognitive impairment in schizophrenia [79]. Considering that KMO is already impaired in individuals with schizophrenia (Figure 5A), one may suggest that the inhibition of KAT would increase Kyn availability as a substrate for kynureninase, consequently leading to a robust increase in AA formation [80,81] (Figure 5B). Therefore, the elevation of AA formation might contribute to the nootropic effect of KYN-5356. Such an intervention might be especially effective in the elderly, considering the aging-associated decline of rat liver kynureninase activity [82], and in male schizophrenia patients, given the lower plasma AA levels in males compared to females [61].

### 13.6. Nervus Vagus Stimulation

A significant elevation of plasma AA levels was reported in children with intractable epilepsy treated via vagus nerve stimulation (VNS). AA CSF and plasma levels were significantly higher at the end of the treatment compared with the baseline (*p* = 0.002) in a randomized, active-controlled, double-blind study of forty-one children with intractable epilepsy [83]. Notably, there was no elevation in the levels of other studied kynurenines, such as tryptophan, Kyn, KYNA, 3HK, and xanthurenic acid. In the context of the proposed cascade, the elevation of AA plasma levels might contribute to VNS-induced memory improvements [84].

## 14. Exogenous Agonists of G-Protein Coupled Receptor 109A and Their Nootropic Effect

The upregulation of the formation of endogenous AA, an adaptive mechanism aimed to attenuate the development of cognitive impairment, might be insufficient and require augmentation through additional interventions to support their trajectory. Mega doses of niacin, a GPR109A agonist, decrease blood cholesterol, triglycerides, and FFA levels but induce skin flushing as a common side effect in treated individuals [85]. Notably, niacin, BHB, and BA are not selective agonists for GPR109A and have low affinity for GPR109B [86]. Therefore, high-affinity GPR109A agonists were designed for the treatment of dyslipidemia. However, phase 2 clinical trials of two GPR109A agonists, MK-1903 and SCH900271, found that these agents lower FFA plasma levels (suggesting the inhibition of myelin degradation) but do not affect cholesterol and triglyceride levels. Further studies on the effects of niacin in GPR109A knockout mice revealed that GPR109A mediates niacin’s ability to lower FFA levels but not cholesterol and triglycerides levels [87]. Similar results were reported in clinical trials with another GPR109A agonist, GSK256073 [88]. The exploration of nootropic activity in synthetic GPR109A agonists holds significant translational potential, considering that these compounds have already been studied in phase 1 and 2 clinical trials.

## 15. Additional Considerations

### 15.1. AA and Diet

In the context of the proposed cascade, the downregulation of GPR109A (which disinhibits cPLA2 activity) contributes to the development of cognitive impairment, while the activation of GPR109A (which inhibits cPLA2) exerts nootropic effects (Figure 1 and Figure 3). Notably, 11 weeks of high-fat diet exposure downregulates GPR109A gene expression in the epididymal fat pads of male C57BL/6 mice [35]. Therefore, diet-induced obesity might contribute to the association between obesity and the risk of cognitive impairment. As mentioned earlier, concentrations of GPR109A agonists, such as BHB, a ketone body produced by hepatocytes, are not sufficient to activate GPR109A under physiologic conditions. However, short-term starvation activates GPR109A due to elevated endogenous BHB levels through ketosis [86]. Furthermore, hyperglycemia (i.e., an increase in glucose levels in the medium) elevates cPLA2 activity in cultured rat and human vascular smooth muscle cells, as well as in rat capillary endothelial cells [27]. These results suggest that insulin resistance may contribute to cognitive impairment via cPLA2 activation and the subsequent degradation of myelin.

### 15.2. Food Supplements

#### Citrus Honey

Higher levels of methyl-anthranilate were found in citrus honeys compared to non-citrus honeys [89,90]. Notably, KYNA, another downstream catabolite of Kyn, was found in relatively high concentrations in honeys, especially in chestnut honey [91,92].

As mentioned above, pyridoxal phosphate (vitamin B6) is a co-factor for kynureninase that is very sensitive to vitamin B6 deficiency. Vitamin B6 was the only B vitamin predictive of cognitive decline (assessed via the Mini-Mental State Examination). A lower dietary intake of vitamin B6 (0.9–1.4 mg/day) was associated with a 3.5–4-fold greater risk of cognitive decline in a 4-year follow-up study of 215 participants 73.4 ± 7.1 years of age [93]. Considering that the addition of vitamin B6 resulted in a 5-fold increase in kynureninase activity in rat liver homogenate [94], it is possible that the nootropic effect of vitamin B6 is mediated via the upregulation of AA formation.

### 15.3. Sex-Specific Elevation of AA

The present review reveals intriguing associations between plasma AA levels and clinical psychopathology in schizophrenia and preclinical AD. In both cohorts, such an association was only observed in female subjects [63,64]. Furthermore, sodium benzoate improves cognition only in female patients with late-stage AD [95]. The inhibition of KAT by estrogens [96] might contribute to female-specific AA elevation, considering that Kyn is a common substrate for both kynureninase and KAT, while KMO activity is downregulated in individuals with schizophrenia (Figure 5B). Furthermore, in a study of thirty-four individuals with AD (20 females and 14 males), a positive Spearman rank correlation between the Global Deterioration Scale (GDS) scores and blood levels of cortisol, an inducer of TDO, an enzyme that catalyzes tryptophan conversion into Kyn, an immediate AA precursor, was observed only in female individuals with AD after the administration of 0.5 mg of dexamethasone [97]. The female-specific elevation of AA formation might contribute to postmortem, and imaging findings that show that human females reach higher myelination levels earlier than males in multiple brain regions [98].

### 15.4. AA Elevation as Early Biomarker of Cognitive Impairment

Identifying early symptoms of brain-related diseases is crucial for timely intervention and treatment [99]. Early, non-invasive biomarkers of cognitive impairment are essential for identifying individuals at risk and determining the optimal timing for preventive interventions [100]. As mentioned above, elevated plasma AA concentrations were detected in cognitively normal individuals who later developed dementia, and plasma AA elevation was correlated with a high neocortical Aβ load in preclinical AD in female (but not male) cognitively normal individuals. Therefore, blood AA levels should be further studied as potential early biomarkers of cognitive impairment and indicators for the timing of preventive interventions.

### 15.5. AA Elevation Is an Adaptive (Compensatory) Response Aimed at Attenuating Cognitive Impairment

Considering that the upregulation of AA formation precedes the development of clinical symptoms of AD and is associated with the improvement of episodic and working memory [65], the present review suggests that the upregulation of the formation of AA, a putative endogenous GPR109A agonist, is a compensatory (adaptive) response aimed at attenuating the development of cognitive impairment in schizophrenia, MCI, and AD (Figure 3). However, adaptive mechanisms might not be sufficient to halt the pathological process. As was noted earlier, Kyn is the common substrate for the formation of AA, KYNA, and 3-HK. Kynureninase has lower affinity to Kyn than KAT and KMO, enzymes that catalyze Kyn formation into KYNA and 3HK, respectively. The inhibition of KMO results in a 7-fold increase in rat brain AA activity [53], and KAT inhibition results in a 12-fold increase in the urine AA excretion in baboons [101]. However, the elevation of AA blood levels in individuals with schizophrenia was, at most, around 30% [62] and was not found in AD [102,103]. Therefore, clinical data suggest only the partial activation of kynureninase activity in individuals with schizophrenia and AD. Future studies should compare circulating AA levels in individuals with MCI and AD.

## 16. Conclusions and Future Directions

### 16.1. Upregulated Formation of AA and Cognitive Impairment

The present review suggests a new target for nootropic intervention, i.e., the activation of endogenous AA formation. This suggestion warrants clinical trials of pegylated kynureninase, pegylated INF-alpha, and VNS as nootropic interventions.

### 16.2. GPR109A and the Pathogenesis of Cognitive Impairment

The major conclusion of the present review is that the AA–GPR109A–cPLA2–myelin–cognition cascade is a new target for the prevention and treatment of cognitive impairment in neurodegenerative disorders. The nootropic effect (if any) of GPR109A agonists implies, but not necessarily proves, a causative link between GPR109A downregulation and cognitive impairment in schizophrenia. A genome-wide study suggested an association between genetic mutations in GPR109A and a diminished skin flush response to niacin, a feature observed in a subgroup of schizophrenia patients [16]. However, GRP109A expression has not been assessed in individuals with cognitive impairment. The expression of GPR109A may help identify a new endophenotype of schizophrenia [104,105]. These suggestions require further preclinical and clinical studies, especially considering the paucity of data on AA formation and cognition.

### 16.3. Repurposing Exogenous GPR109A Agonists as Nootropic Medications

The present review suggests that the upregulation of the formation of AA, a putative endogenous GPR109A agonist, is a sex-specific adaptive response aimed at preventing the development of cognitive impairment through the activation of microglial GPR109A, the inhibition of cPLA2, and the attenuation of myelin degradation. Notably, adaptive mechanisms may fail and require substitution (or replacement) with additional interventions that support their trajectory. In the context of the proposed cascade, preventing or treating cognitive impairment will require repurposing exogenous GPR109A agonists, originally designed as AA derivatives, as nootropic agents. What makes this proposal particularly promising is that exogenous GRP109A agonists, initially designed for the treatment of hyperlipidemia, could potentially be repurposed for treating cognitive impairment in neurodegenerative disorders.

## Data Availability

No new data were created or analyzed in this study. Data sharing is not applicable to this article.

## Abbreviations

AA	anthranilic acid
Aβ	amyloid-β
AD	Alzheimer’s dementia
BA	butyric acid
BHB	R-β-hydroxybutyric acid
cPLA2	cytosolic phospholipase A2
FFA	free fatty acids
GPR109A	G-protein coupled receptor 109A
IDO	indolamine 2,3 dioxygenase
IFN γ	interferon gamma
KAT	kynurenine aminotransferase
KMO	kynurenine 3-monooxygenase
Kyn	kynurenine
KYNA	kynurenic acid
MCI	mild cognitive impairment
NMDAR	N-methyl-D-aspartate receptor
TDO	tryptophan 2,3 dioxygenase 2
VNS	vagus nerve stimulation
